# Pyro-Electrification of Freestanding Polymer Sheets: A New Tool for Cation-Free Manipulation of Cell Adhesion *in vitro*

**DOI:** 10.3389/fchem.2019.00429

**Published:** 2019-06-19

**Authors:** Romina Rega, Oriella Gennari, Laura Mecozzi, Vito Pagliarulo, Martina Mugnano, Emilia Oleandro, Filomena Nazzaro, Pietro Ferraro, Simonetta Grilli

**Affiliations:** ^1^Institute of Applied Sciences and Intelligent Systems, National Research Council (CNR-ISASI), Pozzuoli, Italy; ^2^Department of Mathematics and Physics, University of Campania “L. Vanvitelli”, Caserta, Italy; ^3^Institute of Food Sciences, National Research Council (CNR-ISA), Avellino, Italy

**Keywords:** dipoles orientation, pyro-electrification, pyroelectric effect, lithium niobate, cell patterning and manipulation, bacteria biofilm

## Abstract

Localized electric fields have become, in recent years, a source of inspiration to researchers and laboratories thanks to a huge amount of applications derived from it, including positioning of microparticles as building blocks for electrical, optical, and magnetic devices. The possibility of producing polymeric materials with surface charge thus opens new perspectives for applications where process simplicity and cost-effectiveness of flexible electronics are of fundamental importance. In particular, the influence of surface charges is widely studied and is a critical issue especially when new materials and functional technologies are introduced. Here, we report a voltage-free pyro-electrification (PE) process able to induce a permanent dipole orientation into polymer sheets under both mono- and bipolar distribution. The technique makes use of the pyroelectric effect for generating electric potentials on the order of kilovolts by an easy-to-accomplish thermal treatment of ferroelectric lithium niobate (LN) crystals. The PE allows us to avoid the expensive and time-consuming fabrication of high-power electrical circuits, as occurs in traditional generator-based techniques. Since the technique is fully compatible with spin-coating-based procedures, the pyro-electrified polymer sheets are easily peeled off the surface of the LN crystal after PE completion, thus providing highly stable and freestanding charged sheets. We show the reliability of the technique for different polymers and for different applications ranging from live cell patterning to biofilm formation tests for bacteria linked to food-processing environments.

## Introduction

The possibility of tailoring the material surface properties has allowed an increasingly active field for a wide variety of researches and applications ranging from materials science, nanotechnology, and electronics to biological and medical systems (Stuart et al., 2010). One possibility for functionalizing surfaces and thus tuning interfacial properties is to use specific chemical treatment or to design and induce the electrostatic charges in specific locations. In particular, materials that show net electrostatic charges have been the key issue for fruitful applications in electronics, mechanics, and biological systems. Supports with localized charges are widely used to control the behavior of thin-film electronic devices (Jacobs and Whitesides, 2001). Moreover, obtaining templates with a pattern of electrostatic charges becomes very useful in applications including sorting and self-assembling of micro- and nano-particles (Palleau et al., 2011; Zhao et al., 2011), macromolecules (Seemann et al., 2007; Zhao et al., 2011; Xi et al., 2012), or other building blocks (Cole et al., 2010). Recently, the programming and patterning of electric charges have been widely demonstrated by simple and effective processes in ferroelectric substrates (Grilli et al., 2008b; Esseling et al., 2013; Gennari et al., 2013; Carrascosa et al., 2015; Chen et al., 2016) devoting much attention to the possibility of using polymeric materials thanks to their ability to be economically produced on a large scale and with the further advantage of flexible thin-film technology. The techniques for charging polymeric surfaces are numerous and are quite well-established. The most common approaches require an external voltage source as in the case of contact poling (Hill et al., 1994) or corona poling (Rychkov et al., 2011). However, these techniques present severe limitations. In the case of contact poling, a large charge injection could produce a detrimental dielectric breakdown of the films (Hill et al., 1994; DeRose et al., 2006), while in the case of corona poling, the good homogeneity of the polarization is not guaranteed due to the difficult control of the high field intensity required. In addition, the poled films present often several surface damages due to various reactive and energy species, such as ozone or nitrogen oxides, that are produced by the corona discharge (Sprave et al., 1996).

Here, we propose a simple and voltage-free process based on pyro-electrification (PE), capable of producing polar orientation and 2D polar patterning in freestanding polymer sheets. Compared to conventional electrification techniques, the PE is electrode-free and is able to produce high voltages by a simple thermal treatment in ferroelectric crystals of lithium niobate (LN), thus simplifying the whole process significantly. Moreover, we can produce both homogeneous and bipolar charge distributions simply by using one-domain or homemade periodically poled LN crystals obtaining pyro-electrified polymer sheets with a net surface charge. Recently, we investigated the presence of such charge by analyzing the second harmonic signal and by observing the adhesion and spreading of eukaryotic cells on PE polymers that would be otherwise cytophobic surfaces (Rega et al., 2016a,b; Lettieri et al., 2017). Moreover, we evaluated *in vitro* the ability of bacteria to form biofilms very rapidly (Gennari et al., 2018).

It is recognized that the adhesion and proliferation of different types of cells on polymeric materials depend on surface characteristics and can be significantly influenced by the surface charge on that material (Robertus et al., 2010; Li et al., 2018; Liu et al., 2018; Ma et al., 2019).

Polystyrene (PS) and poly (methyl methacrylate) (PMMA) are materials typically used in labware equipment. They are cytophobic and require a chemical or physical treatment to promote cell adhesion. The most common treatments make use of sulfuric acid, or chromic acid, which can produce different kinds of functional groups on the surface, such as sulfonate, hydroxyl, or carboxyl. Another family of techniques makes use of protein adsorption, such as fibronectin, laniline, and Arginylglycylaspartic acid (RGD) peptide that chemically mimic the extracellular microenvironment and alter the structure of the surface. On the other hand, physical treatments able to activate the surface of traditional cytophobic surfaces by mild oxidation involve ultraviolet light or corona discharge stimulation. Regardless of the method, the cell adhesion promotion requires an electrostatically charged surface. All of these treatments are well-established but are laborious and expensive with additional drawbacks related to potentially polluting materials.

Here, we show the possibility of obtaining charged polymeric surfaces capable of interacting electrostatically with cell cultures under safe conditions by an easy and economical procedure, tracing the route as an alternative tool for all those applications in which the use of chemical agents or complicated physical treatments can be detrimental for both environment and cell cultures.

## Materials and Methods

The ferroelectric LN is a rhombohedral crystal belonging to the point group 3 m that exhibits pyroelectricity at room temperature. The spontaneous polarization *P*_s_ changes according to Δ*P*_i_α*p*_i_Δ*T*, where *P*_i_ is the coefficient of the polarization vector, *p*_i_ is the pyroelectric coefficient, and Δ*T* is the temperature variation. At room temperature, the equilibrium condition makes the charge of the spontaneous polarization *P*_s_ to be balanced fully by the environmental screening charges, and no electric field exists (Grilli et al., 2008a). The temperature variation changes the polarization magnitude and perturbs this equilibrium, causing a lack or excess of surface screening charge (Bhowmick et al., 2017). As a consequence, an electrostatic state appears and generates a high-intensity electric field at the crystal surface (Bhowmick et al., 2017) with mono- or bipolar properties depending on the nature of the LN crystal, mono-domain in the first case and periodically poled (PPLN) in the second one. The PPLN crystals were homemade by standard electric field poling onto photoresist patterned samples (Detrait et al., 1998; Huang et al., 2012; Pagliarulo et al., 2018) and consisted of an array of ferroelectric domains with opposite polarization. The pyroelectric effect generates an array of surface charges with opposite sign, following the pattern of the reversed domains. Recently, we demonstrated for the first time the possibility of using the pyroelectric effect for a wide variety of applications ranging from biological to soft matter manipulation application (Ferraro et al., 2010; Mecozzi et al., 2016, 2017; Rega et al., 2019).

The PE exploits this electric field during an appropriate thermal treatment. The PE induces a permanent orientation of the dipoles when exceeding the glass transition temperature of the polymer, namely, when the dipole molecules can be easily oriented. This technique guarantees simplicity and cost-effectiveness since only a polymeric solution is needed, spin coated on an LN crystal and thermal treated on conventional hotplates. The fundamental role is played by the LN crystal since it supports, heats, and polarizes the polymer layer, which, at the end of the process, is easily removed, obtaining a freestanding charged sheet.

## The Pyro-electrification

**Figure 1:**
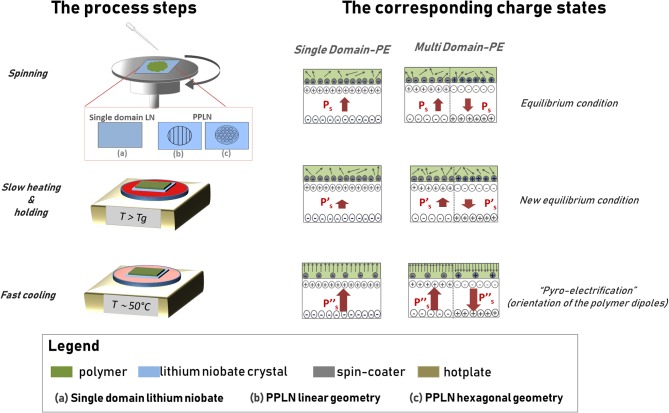
Schematic view of the pyro-electrification (PE) technique. The column on the left shows the view of the process steps, while the column on the right shows the view of the corresponding charge states in the polymer sheet and in the lithium niobate (LN) crystal.

Figure 1 shows the schematic view of the procedure.

The LN crystal sample is spin coated with a polymer solution at room temperature and then heated by a digitally controlled hotplate according to a thermal treatment based on three steps identified here by the following labels: slow heating (Stuart et al., 2010), holding (Jacobs and Whitesides, 2001), and fast cooling (Palleau et al., 2011). The slow heating induces a temperature rise from room temperature (T_*i*_) up to the glass transition temperature of the polymer (T_*f*_) at a rate of 1°C/min. The holding step keeps the sample at the final temperature T_*f*_ for 10 min. In the fast cooling step, the polymer-coated crystal is moved to a second hotplate set at a lower temperature T_*l*_ ~50°C. The slow rate of temperature increase in the first step and the holding state are of crucial importance for keeping the electrostatic equilibrium between the slight polarization change of the LN and the screening charges on the surface, prior to reaching the glass transition of the polymer. Once this state is reached, the polymer becomes very amorphous and the dipole molecules can orient easily under the action of the strong electric field generated onto the surface of the crystal by the pyroelectric effect. The monotonic cooling step is necessary to provide large Δ*T* that generates a steady and strong electric field capable of poling the polymer and, simultaneously, freezing the poled state into the polymer itself. The poled polymer layer can be peeled off from the surface of the crystal proving a freestanding sheet with the same polarization orientation of the LN crystal that drove the PE process.

The technique can be performed under two different configurations and with different polymer solutions. The single-domain PE (SD-PE) uses a single domain crystal, while the multi-domain PE (MD-PE) makes use of a periodically poled crystal PPLN with different geometries. In the case of the SD-PE configuration, the polymer layer exhibits a polarization charge with single orientation, while in the case of the MD-PE configuration, the polymer shows the pattern of domains with reversed polarization according to the ferroelectric domain pattern of the driving PPLN crystal. The driving crystal can be re-used indefinitely after appropriate solvent cleaning.

## The Polymer Solutions

Solid-state polymers were used as received, without further purification (Sigma-Aldrich, Milan, Italy). Polysulfone (PSU) transparent pellets (*M*_w_ 35,000) were dissolved at 80% w/w in anisole and stirred at 70°C for 3 h. PS powder (*M*_w_ 350,000) was dissolved at 60% w/w in anisole and stirred at 70°C for 6 h. PMMA (*M*_w_ 996,000) was dissolved at 15% w/w in anisole and stirred at 70°C for 3 h. The resulting polymer solutions of PSU, PS, and PMMA were stored at 4°C.

## The Periodically Poled LN Crystals

The LN crystals were bought from Crystal Technology Inc., Palo Alto, California, in the form of both sides polished into 500 μm-thick c-cut 3-inch. wafers and were cut into square samples (2 × 2 cm^2^) by a standard diamond saw. The PPLNs were obtained by standard electric field poling onto photoresist-patterned samples (Huang et al., 2012; Bhowmick et al., 2017; Pagliarulo et al., 2018). Two geometries were considered: linear (period, 200 μm) and square array of hexagons (period, 200 μm).

## The Bare Sheets (Control)

The freestanding PSU, PS, and PMMA bare sheets were obtained by spin coating a 2 × 2 cm^2^ sized glass coverslip at 4,000 RPM for 2 min with the polymer solution and by peeling off the slide accurately just after solvent evaporation.

## The SH-SY5Y Human Neuroblastoma and the NIH-3T3 Mouse Embryonic Fibroblast Cells

The SH-SY5Y and NIH-3T3, cells lines were purchased from European Collection of Authenticated Cell Cultures (ECACC) (Sigma-Aldrich, Milan, Italy). They were routinely grown in Dulbecco's modified Eagle's medium (DMEM) containing 4.5 g/L D-glucose and supplemented with 2 mM L-glutamine, penicillin (100 units/ml), and streptomycin (100 μg/ml), and containing 20% (v/v) fetal bovine serum (FBS) (GIBCO, Gaithersburg, MD, USA). For the cell culture experiments, the SH-SY5Y and NIH-3T3 were detached by means of Trypsin/Ethylenediaminetetraacetic acid dipotassium salt dihydrate (EDTA) solution (Sigma, Milan, Italy), resuspended in DMEM−20% FBS, seeded at a concentration of 1.0 × 10^5^ cells/ml on the MD-PE sheets (immersed in DMEM medium at 37°C for 1 h prior to use), and then incubated into conventional 30 mm-diameter Petri dishes at 37°C and in a saturated humidity atmosphere containing 95% air and 5% CO_2_. Cells were allowed to grow in DMEM−20% FBS on different substrates for 24 h. Cell adhesion and spreading were observed over 24 h under a standard inverted optical microscope (AxioVert, Carl Zeiss, Jena, Germany).

## Immunofluorescence

The cells were cultured 24 h on the surface of interest and then fixed by standard procedures. The cells were then stained by Alexa Fluor 488 phalloidin and by blue fluorescent Hoechst 33,342 dye and trihydrochloride trihydrate (Molecular Probes Invitrogen) for visualizing nuclei and actin filaments.

## Bacterial Strain and Culture Conditions

In the present study, we used the Gram-positive Listeria innocua (strain DSMZ 20649) provided by the Deutsche Sammlung von Mikroorganismen und Zellkulturen, Braunschweig, Germany. The bacteria strains were plated and incubated on Luria-Bertani (LB) agar plates (10 g/l NaCl, 10 g/l tryptone, 5 g/l yeast extract, and 15 g/l agar, Thermo Fisher Scientific). One day before the experiments, a single bacterial colony was picked up and cultured in LB broth medium at 37°C in a shaker incubator at 225 RPM for 16–18 h to achieve saturation conditions. A 1:5 volumetric dilution of the cell culture was then grown in LB until reaching the log phase. Then, the growth was stopped and bacteria were harvested by centrifugation at 7.200 g (Beckman Coulter tj-25 centrifuge, California, USA) for 10 min in order to separate the cells from the medium. Sterilized LB broth was measured (3 ml) into sterile tubes. The bacteria concentration was evaluated by the spectrophotometric measurement (Bio-Rad SmartSpecTM Plus Spectrophotometer, California, USA) of the suspension absorbance at 600 nm (optical density at 600 nm, i.e., OD_600_), considering that 8 × 10^8^ cells/ml have an OD_600_ = 1.

## Microtiter-Plate Test

In order to quantify the biofilm formation, we use the crystal violet assay (Stepanović et al., 2000). This technique involves fixing the bacterial film with methanol, staining with crystal violet, releasing the bound dye with 33% glacial acetic acid, and measuring the optical density (OD) of the solution at 600 nm by spectrophotometric measurement (Bio-Rad SmartSpecTM Plus Spectrophotometer, CA, USA).

## The Viability Test

The viability of the bacterial strain (*L. innocua*) was tested through the live/dead viability/cytotoxicity assay kit (Live/Dead *Bac*Light bacterial viability kit, Thermo Fisher Scientific, Waltham, MA, USA). The easy-to-use live/dead kit is utilized for monitoring the viability of the bacterial populations as a function of the sheet integrity of the cell. The live/dead BacLight Bacterial Viability Kits utilize mixtures of our SYTO® 9 green-fluorescent nucleic acid stain and the red fluorescent nucleic acid stain, propidium iodide. These stains differ both in their spectral characteristics and in their ability to penetrate healthy bacterial cells. Cells with a compromised sheet that are considered to be dead or dying will stain red, whereas cells with an intact sheet will stain green. The cells were incubated on each substrate for 24 h. After incubation, each substrate was immersed in 8 μl of 1,000-fold diluted live/dead kit solution and was incubated for 15 min in the dark. The fluorescence micrographs were acquired by an inverted laser scanning confocal microscope (Zeiss LSM 700, Germany), equipped with a 20 × objective.

## Results and Discussion

**Figure 2:**
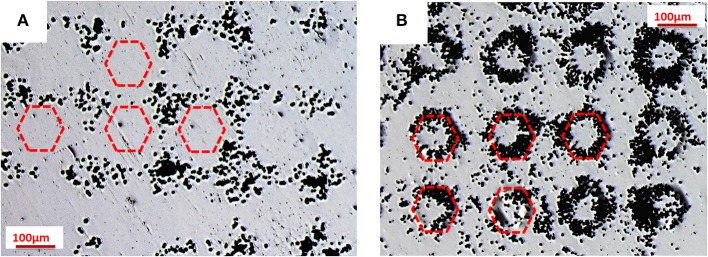
Optical microscope images of decoration technique: toner distribution on two polystyrene (PS) sheets after pyro-electrification (PE) by a periodically poled lithium niobate (PPLN) crystal, where the hexagons exhibit **(A)** positive and **(B)** negative charge polarity. The hexagons are indicated by the schematic drawings with the red dotted line.

Two PMMA sheets were subjected to PE by using a PPLN crystal (see Figure 1 for the multi-domain procedure). A faster test to verify the presence of the charges on the PE sheet is the decoration technique. The polymer solution was spun on one face of the crystal and, in the second experiment, on the other face of the PPLN. As a consequence, we produced two freestanding PS sheets with a bipolar polarization pattern following the pattern of reversed domains of the PPLN. The hexagonal regions of the PS sheets exhibited positive and negative polarity, respectively. Figures 2A,B shows the typical optical microscope images of these two PS sheets after gentle sprinkling of toner dust on the surface.

These images show clearly that the particles of toner, bringing a negative charge, are attracted electrostatically by the hexagonal regions (positive charge) in the first case (see Figure 2A) and by the surrounding regions (positive charge) in the second case (see Figure 2B), thus demonstrating the non-negligible electric field generated on the surface of the pyro-electrified PS sheets.

We demonstrate here the possibility of using the permanent electric field on the PE sheet for guiding live cell adhesion *in vitro*. PS and PMMA labware equipment have been used for cell culture since about 1965. Many cell types adhere to and move on the surfaces of such materials and present a morphology that is very similar to that seen when the cells are grown on glass. However, it has long been known that these materials must be subjected to a surface treatment to render their surface suitable for cell attachment. Bare PS and PMMA are unsuitable for cell attachment, meaning that cells seeded and incubated onto surfaces made of such polymers cannot find adhesion cues and remain suspended. This has been attributed to the surface chemistry of the materials, and many different specific surface treatments have been made to change the chemistry involved in the non-adhesive nature of these polymers (Klemperer and Knox, 1977; Maroudas, 1977; Grinnell, 1978; Lee et al., 1994; Buttiglione et al., 2007).

**Figure 3:**
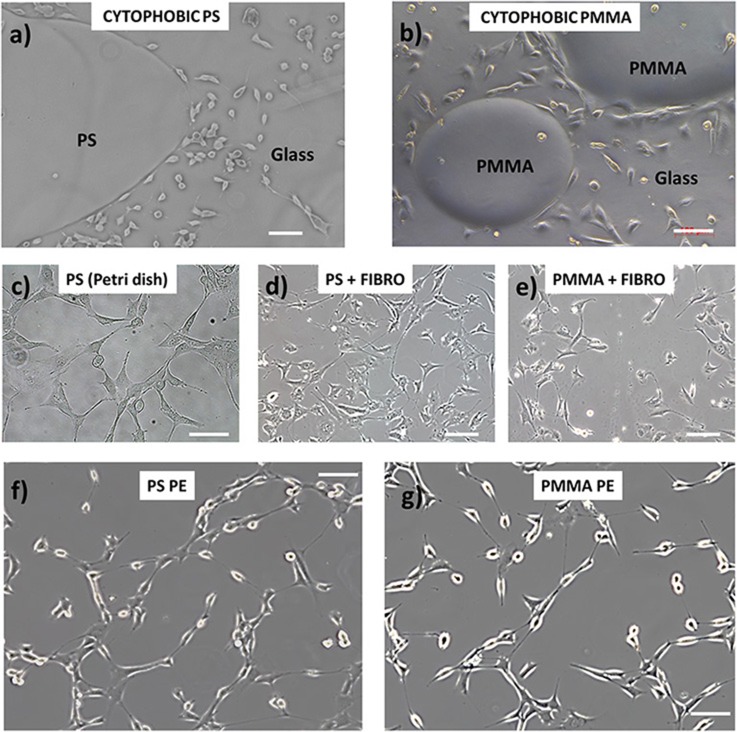
NIH-3T3 cell adhesion behavior on **(a,b)** bare PS and poly(methyl methacrylate) (PMMA) polymer (respectively) spotted on a glass coverslip where it shows the cytophobic behavior of the materials; **(c–e)** conventional PS Petri dish (generally treated with UV light), PS coated with fibronectin protein, and PMMA coated with fibronectin protein, respectively; **(f,g)** NIH-3T3 cells grown on the pyro-electrified PS and PMMA, respectively. Scale bar, 100 μm.

Here, we demonstrate the cytophilic capability of PE polymer for cultured NIH-3T3 cells (see Materials and Methods for details) on PS and PMMA sheets pyro-electrified on a single domain crystal. Figure 3 shows the optical microscope images of the sheets after 24 h incubation with NIH-3T3 cells. In particular, the image in Figures 3a,b show the cytophobic behavior of bare PS and PMMA polymer spotted on a glass coverslip where it is clearly seen that the cells are not able to adhere and spread. These materials are often treated in a chemical or physical way in order to permit cell adhesion and spreading. Figures 3c–e show the adhesion of NIH-3T3 cells on a conventional PS Petri dish (generally treated with UV light), PS coated with fibronectin protein, and PMMA coated with fibronectin protein, respectively. Figures 3f,g show the NIH-3T3 cells grown on the pyro-electrified PS and PMMA, respectively.

On substrates treated with fibronectin, the cellular conformation and their spatial distribution are characterized by a more compact organization and a more spread cellular shape, indicating a stronger cell–cell interaction than the interaction with the adhesion surface. The cellular distribution on the pyro-electrified sheets shows, on the other hand, an elongated shape of the nuclei and cellular filopodes, typical of cells polarized along one preferential direction. This characteristic is an indication of a strong interaction with the contact surface, which is predominant with respect to the cell–cell interaction.

Moreover, as already shown in our previous paper (Rega et al., 2016a,b), it is possible to have a cell pattern configuration when seeded on sheets pyro-electrified by a multi-domain crystal with a bipolar configuration of the charge distribution on the sheet surface. Here, we demonstrate the reliability of the technique with other periods and different concentrations of the polymer.

**Figure 4:**
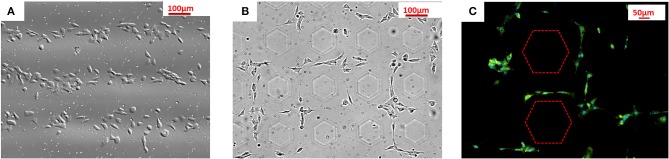
SH-SY5Y cell adhesion behavior on **(A)** PS sheet pyro-electrified linearly at 200 μm; **(B)** cell patterning on PMMA (15% in anisole w/w) sheet pyro-electrified by an array of hexagons at 200 μm; **(C)** immunofluorescence images of cells cultured after 24 h incubation on PS sheets pyro-electrified by an array of hexagons at 200 μm. The dashed lines correspond to the boundaries between regions with opposite polarities.

Figure 4A shows that SH-SY5Y cells adhered selectively on the positive region of PS sheet with bipolar domains having linear geometry with a 200 μm period, while Figure 4B corresponds to a PMMA (15% in anisole w/w) sheet, with bipolar domains having two-dimensional distributions at a 200 μm period. Moreover, in order to better elucidate the material–cytoskeleton cross-talk during adhesion, we fixed the cells after 24 h incubation on PS sheets pyro-electrified by PPLN crystals with hexagonal domains, and we performed immunofluorescence reactions (Figure 4C) (see Materials and Methods for details). The cells appear clearly to adhere selectively on the regions with positive polarity in the case of linear domains as well as in the case of two-dimensional domains. In fact, the cells bring a negative net charge on the external sheet (Ohgaki et al., 2001) and, as a consequence, are attracted electrostatically by the regions of the sheet exposing a net positive polarization charge.

The PE allows us to achieve cell patterning results through a cation-free method, avoiding time-consuming and expensive lithographic-based procedures and moreover directly onto freestanding, and cheap polymer sheets. The relatively easy-to-accomplish procedure, which makes use simply of LN crystals and conventional hotplates, would be easily implementable in biology laboratories for routine cell biology experiments where selective cell adhesion configurations are of crucial importance. For example, in the case of electric-field-sensitive cells, we envisage the possibility of using PE for developing pre-defined neuronal networks for deep studies on their physiology and morphology behavior.

Recently, we demonstrated the possibility of using pyro-electrified sheets and fibers for rapid and reliable “biofilm electrostatic test” (Gennari et al., 2018) of *Escherichia coli* and *Staphylococcus epidermidis*, with potential applications in the field of biomedicine. In fact, the rapid biofilm formation promoted by our sheets would have a significant impact on health service when a fast response to an antibiogram test can save lives. Here, we show how the pyro-electrified sheets promote a rapid biofilm formation also in the case of *L. innocua*, a non-pathogenic species closely related to *L. monocytogenes* (Scifò et al., 2009; Tajkarimi et al., 2016; Jeon et al., 2018; Sheng et al., 2018). We use an SD-PE sheet as a tool for simple, rapid, and cost-effective evaluation of biofilm formation, through the electrostatic interaction of planktonic bacteria with a pyro-electrified carrier (Asadishad et al., 2011; Gennari et al., 2018). The fact that biofilms have a multidisciplinary impact that includes environmental, industrial, and clinical characteristics, and that over 60% of all human bacterial infections and up to 80% of all chronic infections are related to bacterial biofilms, is of fundamental importance in assessing how different environmental factors may affect the bacterial vitality. The cellular mechanisms underlying microbial biofilm formation and behavior are beginning to be understood and are targets for novel specific intervention strategies to control bacteria colonies formation in different fields and in particular for the food-processing environment (Dickson and Koohmaraie, 1989; Sahm et al., 1994; Linke and Goldman, 2011; Bianco et al., 2017; Bruslind, 2017; Mandracchia et al., 2019).

**Figure 5:**
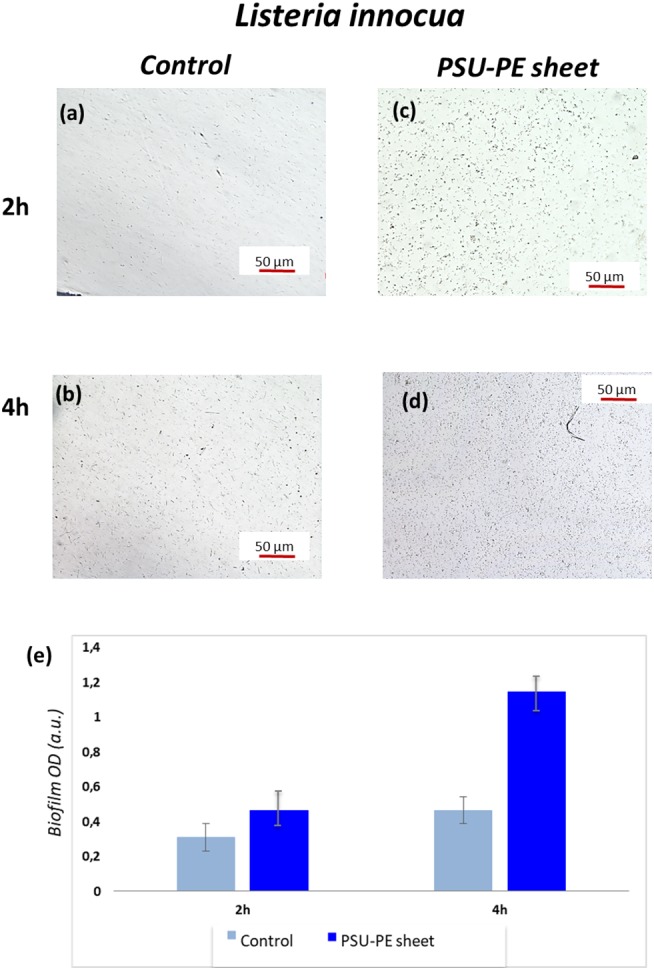
**(a,b)** Optical microscopy images of *L. innocua* bacterial adhesion at two different time points on control and **(c,d)** on the polysulfone pyro-electrified (PSU-PE) sheet; **(e)** histogram of bacteria biofilm OD (optical density) on the control and the PSU-PE sheet at two different time points.

Here, we demonstrate that SD-PE carrier provides a polarization field able to immobilize the *L. innocua* bacteria and test their ability to form live biofilms within 2 h, avoiding time-consuming and laborious incubations and/or intermediate chemical treatments. PSU sheets (about 100 μm thickness and 2 × 2 cm^2^ sized) were produced: (Stuart et al., 2010) a bare sheet that represents the control and (Jacobs and Whitesides, 2001) polysulfone pyro-electrified (PSU-PE), where the positive side was in contact with the bacterial suspension. These sheets were incubated at different times (2 and 4 h) at 37°C in two different Petri dishes (35 mm) covered with 1 ml of *L. innocua* bacterial suspension (Gram-positive) and 2 ml of phosphate-buffered saline (PBS). The control and the SD-PE sheets were observed under an optical microscope, and Figure 5 shows the corresponding typical images.

These microscope images show the immobilized and biofilm forming bacteria on the control and PSU-PE sheet at different time intervals. The number of adhesion bacteria on the PSU-PE sheet appeared clearly higher than that on the control at each observation time. These results are confirmed by the quantitative evaluation of biofilm formation obtained by the microtiter-plate test (see Materials and Methods for details). The diagram in Figure 5e shows results averaged over five measures of biofilm OD (optical density) formation and shows evidence of the larger population of bacteria on the PE sheet.

**Figure 6:**
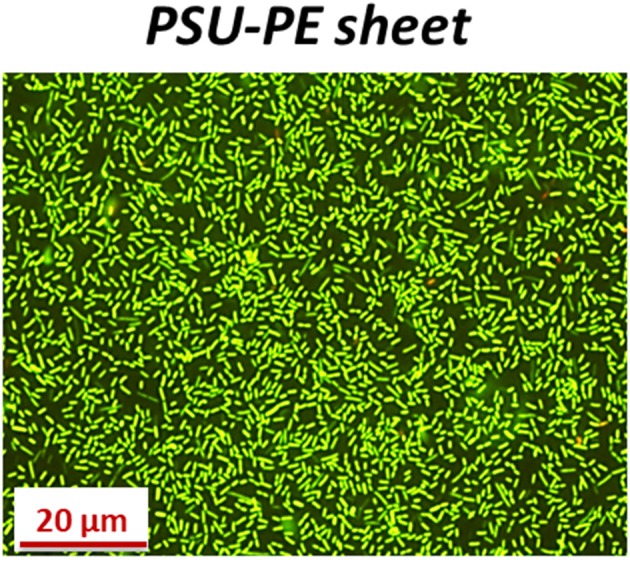
Fluorescence microscopy images of *L. innocua* forming biofilms onto the PSU-PE sheet after 24 h incubation and live/dead staining.

To verify the viability of the biofilms, the PSU-PE sheet was mounted onto a glass slide in a well after 24 h incubation with planktonic bacteria in order to perform the reaction with the live/dead staining kit (see Material and Methods for details). After 24 h incubation, we found that the viability of the high-density biofilms formed onto the PSU-PE sheet was clearly evident (Figure 6). The PSU-PE sheet immobilized planktonic bacteria more rapidly than the control and favored biofilm formation without damaging the bacterial cytomembrane even after 24 h incubation, thus demonstrating biocompatiblity.

**Figure 7:**
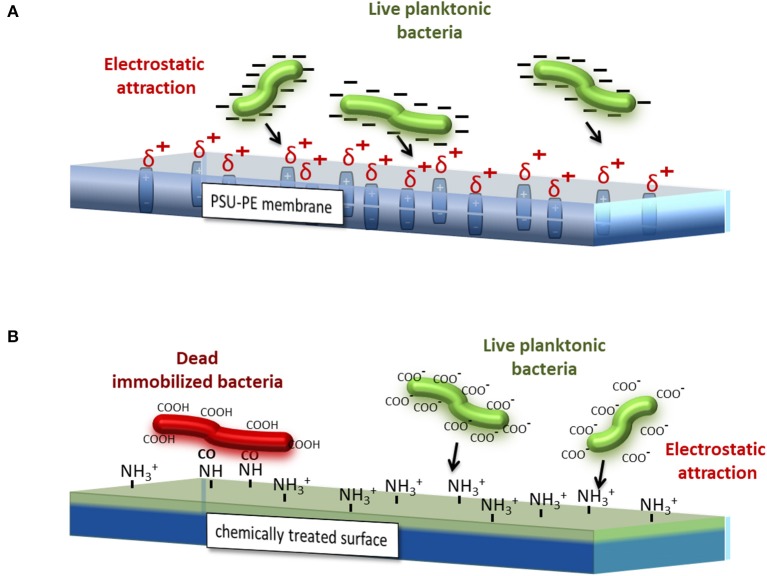
Simplified schematic views of the different interaction mechanisms between bacteria, the cytomembrane, and **(A)** the PSU-PE sheet and **(B)** a generic chemical-coated sheet.

On the contrary, chemical coatings could immobilize planktonic bacteria, but could even destroy the bacterial cytomembrane causing their death. Cationic surfaces are obtained usually by the covalent attachment of different chemical compounds, such as quaternary ammonium organo-silanes (Isquith et al., 1972; Gottenbos et al., 2002; Li et al., 2006), antimicrobial peptides (Gabriel et al., 2006; Gao et al., 2011), polyethylenimines (PEI) (Lin et al., 2003), and many others. The mechanism of interaction, as already explained in Gennari et al. (2018), is schematized in Figure 7. The PE process provides a sheet (in this case, the PSU-PE sheet) with δ^+^ charge due to the permanent dipole generated during the process. Those charges attract the negative net charge onto the bacterial cytomembrane (COO^−^ groups), thus leading to bacteria immobilization and promoting the biofilm formation. In contrast, the cationic groups NH3^+^, generally formed on the chemically treated surface, form an electrostatic bond with the COO^−^ groups onto the cytomembrane of bacteria adhered, thus displacing the divalent cations forming the lipopolysaccharide network. This causes the disruption of the cytomembrane and then microorganism death.

The PE of freestanding polymer sheets could find applications in the food processing field as a quality check tool. The ultimate goal is the development of a rapid and easy PE assay for a quantitative analysis of a food sample, enabling food technologists to detect the contaminant agents in fresh products that represent a serious risk for all the consumers. In fact, it would be desirable having a compact and efficient system that could be used, for example, in an industry outside the laboratory. However, to develop and to design industrial applications for food processing, more data on the interaction between the process and the target organism are required. PE assay approaches will help to close this gap in the future, because they have the potential to be used as a method to control bacteria contamination and to ensure fresh food safety.

## Conclusion

Here, we show an innovative PE technique capable of inducing a permanent dipole charge into freestanding polymer sheets by exploiting the pyroelectric properties of LN crystals, under both single- and multi-domain configurations. The resulting sheets can correspondingly have a mono- and bipolar charge with a magnitude able to selectively promote the adhesion of both eukaryotic and prokaryotic cells. The innovative use of the pyroelectric effect allows us to avoid expensive and time-consuming fabrication of electrodes and high-voltage circuits, since the appropriate thermal treatment of LN generates electrical potentials on the order of kilovolts. The same LN crystal can be used indefinitely for different PE cycles. The technique is free from lithography-based procedures and free from the use of cation-based chemicals, eventually detrimental for specific cell sheet structures. We show how these sheets can be used for cell patterning as well as for rapid biofilm formation. The relatively easy implementation makes PE implementable in a biology laboratory, opening the route to a novel generation of platforms for manipulating cell adhesion *in vitro* through non-invasive polarization charges, with the additional advantage of being flexible, freestanding, and cheap.

## Author Contributions

RR planned, conducted experiments and wrote the manuscript. OG, MM, FN and EO prepared the biological cultures. VP made the PPNL samples. PF and SG supervised the project. All authors discussed the results and contributed to the manuscript.

### Conflict of Interest Statement

The authors declare that the research was conducted in the absence of any commercial or financial relationships that could be construed as a potential conflict of interest.
